# Pictorial essay: Breast USG

**DOI:** 10.4103/0971-3026.63045

**Published:** 2010-05

**Authors:** Geeta Shah, Bijal Jankharia

**Affiliations:** Advanced Radiology Centre, Mumbai, India; 1 Piramal Diagnostics, Jankharia Imaging, Bhaveshwar Vihar, 383, Sardar V P Road, Mumbai, India

**Keywords:** Breast, ultrasound, mammography

## Abstract

USG of the breast is now an established modality. It is used in the characterisation of focal breast lesions as well as in the primary evaluation of mammographically dense breasts. It helps guide biopsies as well. We present a pictorial essay on the role of USG in various breast pathologies

USG has come a long way from being a modality used by the military for detecting flaws in metal to its present status, where high-resolution scans even help us differentiate benign from malignant breast disease.[1]

It is advisable to perform a targeted breast USG whenever there is a palpable or focal mammographic abnormality in the breast. Although USG is not efficacious as a screening modality, combined mammography and USG pick up more cancers than mammography alone.[2]

For USG examination of the breast, a linear-array transducer of at least 7 MHz frequency is required with a machine that has good spatial and contrast resolution. The patient is scanned in the supine position and then in the contralateral oblique position for the axillary and upper outer quadrants. Color Doppler is not very effective but three-dimensional coronal imaging,[3] as well as elastography, may help to avoid unnecessary biopsies in benign-appearing lesions.

Reporting of the USG and mammography findings is facilitated with the breast imaging reporting and data system (BIRADS) proposed by the American College of Radiology (ACR), which is available at www.acr.org and in some articles.[4]

## Developmental Breast [Figure 1]

**Figure 1:**
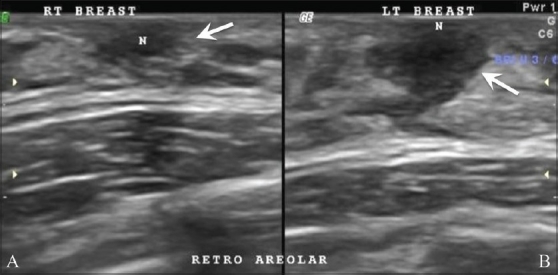
Unilateral early ripening. A 9-year-old girl presented with a tender lump in the left breast. Transverse USG of the right (A) and left (B) breasts shows a subareolar “mass,” (arrows) which represents premature asymmetric ripening

The prepubertal breast has only minimal duct development around the nipples. Sometimes this may be asymmetric and may mimic a subareolar mass (similar to gynecomastia in males). This is called premature asymmetric ripening. It is very important to recognize it because if it is surgically excised by mistake, there will be no breast development on the operated side. The findings are identical to those of asymmetric gynecomastia in males.[5]

## Normal Anatomy [Figure 2]

**Figure 2:**
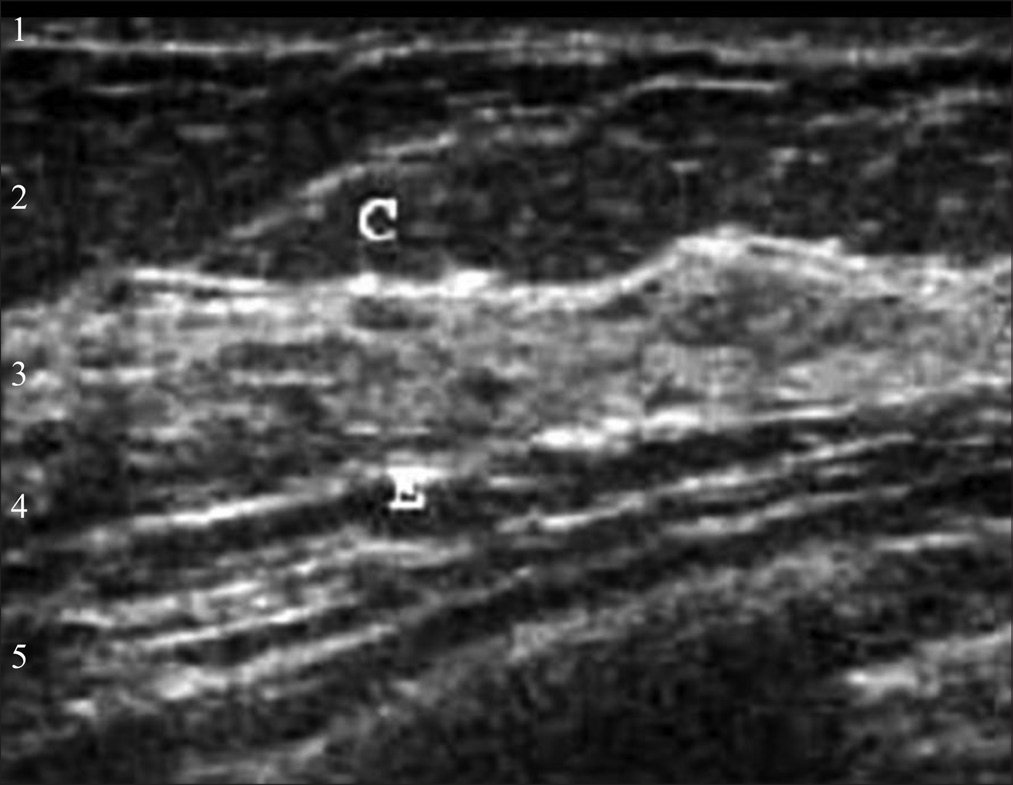
Normal breast anatomy on USG. 1 – hyperechoic skin, 2 – hypoechoic subcutaneous fat, 3 – hyperechoic fibroglandular parenchyma, 4 – hypoechoic retromammary fat, 5 – hyperechoic muscle. C – Cooper's ligaments

The breast has alternate hyperechoic and hypoechoic layers as follows:

1. Skin – hyperechoic
2. Subcutaneous fat – hypoechoic
3. Fibroglandular parenchyma – hyperechoic
4. Retromammary fat – hypoechoic
5. Muscle, mainly the pectoralis major – hyperechoic

Cooper's ligaments are echogenic bands that suspend the breast from the superficial layer of the superficial fascia.

## Three-dimensional Volume USG [Figure 3]

**Figure 3:**
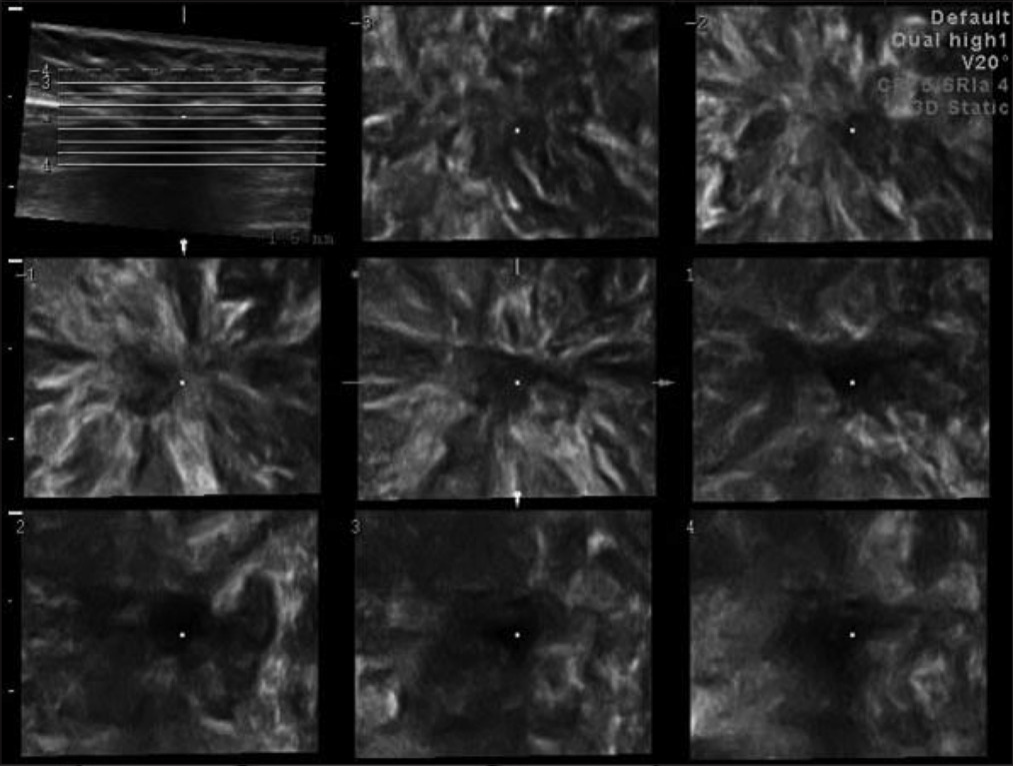
Tomographic USG imaging (TUI) shows a retraction pattern in the coronal plane of a malignant mass

The coronal breast plane is never seen on routine 2D USG. Benign lesions show a compression pattern, whereas malignant lesions show a retraction pattern.

## Cysts [Figure 4]

**Figure 4 (A-D):**
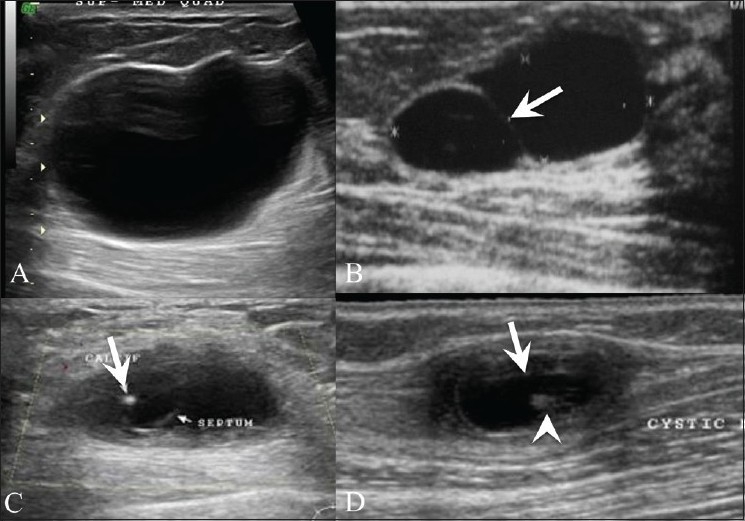
Cysts. Breast USG shows a simple cyst (A), presenting as an anechoic lesion with posterior enhancement and a cyst (B) with a septum (arrow). Breast USG (C) in a 26-year-old lady with a painless palpable lump in her left breast shows a complex cyst, with an eccentrically placed echogenic focus (arrow) representing the scolex of a cysticercus granuloma. She also had a similar swelling in the right upper arm, a USG (D) of which also revealed a cyst (arrow) with an echogenic scolex (arrowhead), within. She was treated with albendazole

Simple cysts in the breast are completely anechoic, with a thin echogenic capsule, posterior enhancement, and thin edge shadowing [Figure 4A]. Complex cysts have intracystic echoes, septae or thick walls, and may be seen in clusters [Figure 4B]. Although this is rare, we have seen a case of breast cysticercosis in a patient who also had a cysticercus granuloma in the biceps tendon [Figure 4C,D]. A significant number of complex cysts, especially those with a solid intracystic mass, may be malignant.[6]

## Intraductal and Intracystic Papillomas [Figure 5]

**Figure 5 (A,B):**
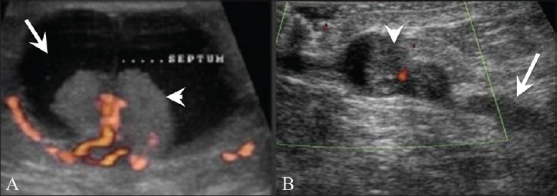
Intracystic (A) and intraductal (B) papillomas. Breast USG with color Doppler (A) in a 40-year-old with a palpable lump and bloody left nipple discharge shows a complex cyst (arrow) with a vascular, intracystic, solid polypoidal mass (arrowhead). A diagnosis of intracystic papillocarcinoma was made on excision biopsy. Breast USG (B) in a 60-year-old patient with a history of left-sided bloody nipple discharge shows a focally dilated duct (arrow) with an intraductal solid echogenic mass (arrowhead)

Papillomas in the breast may be intracystic [Figure 5A] or intraductal [Figure 5B]. They are difficult to differentiate from papillary carcinomas and a biopsy is required for the same. Intraductal papillomas are the most frequent cause of a bloody nipple discharge.

## Fibroadenoma [Figure 6]

**Figure 6 (A,B):**
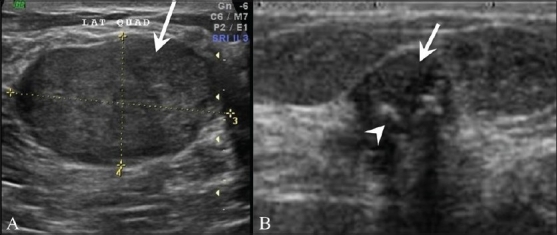
Fibroadenoma. Breast USG in a 25-year-old lady shows a homogenous, hypoechoic, gently lobulated lesion (arrow), suggestive of a fibroadenoma. A degenerating fibroadenoma (arrow) with coarse calcifications (arrowheads) and posterior shadowing from the calcific foci is seen in this breast USG (B)

Fibroadenoma is the most common benign tumor in the breast and is usually seen in young women. It may increase in size during adolescence or pregnancy and lactation, and undergo atrophic changes after menopause. It is usually homogenous, well-circumscribed, hypoechoic, ellipsoid, wider than tall, and may even show posterior enhancement on USG [Figure 6A]. It may undergo calcific degeneration. The calcifications within a fibroadenoma are coarse and may show posterior shadowing [Figure 6B]. Complex fibroadenomas, that is, fibroadenomas with epithelial calcifications, papillary apocrine metaplasia, sclerosing adenosis, and cysts larger than 3 mm, have a higher incidence of transformation into breast cancer.[7]

## Phyllodes Tumors [Figure 7]

**Figure 7:**
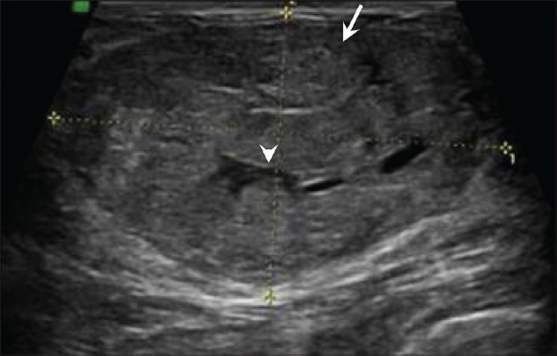
Breast USG in a patient with a rapidly growing lump in the left breast shows a well-defined, gently lobulated, hypoechoic, mildly vascular encapsulated mass (arrow) with multiple, linear, anechoic “clefts” (arrowhead), findings classically seen in a phyllodes tumor. A distinction between benign and malignant phyllodes, however, cannot be made on USG findings

These are rapidly growing, benign-looking lesions with cleft-like cystic spaces and are moderately vascular on USG.[8] They are fibroepithelial tumors that may be benign or malignant. They tend to recur and may rarely metastasize.

## Breast Abscess [Figure 8]

**Figure 8 (A,B):**
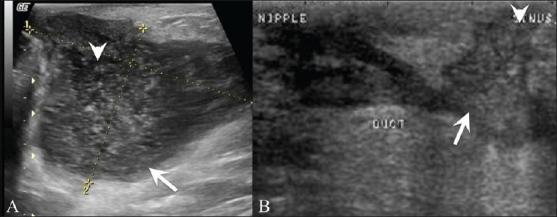
Abscesses. Breast USG (A) in a 30-year-old lactating woman with a painful breast mass, skin erythema, and high-grade fever shows a large heterogeneous, predominantly cystic mass (arrow) with mobile internal echoes (arrowhead), suggestive of a puerperal abscess; this was drained under USG guidance. Breast USG (B) in a patient with a tuberculous breast abscess (arrow) shows a discharging sinus (arrowhead).

Acute abscesses may occur during lactation and are clinically evident [Figure 8A]. In our country, chronic abscesses may be due to tuberculosis and may present as breast lumps with discharging sinuses [Figure 8B].

## Breast Edema [Figure 9]

**Figure 9:**
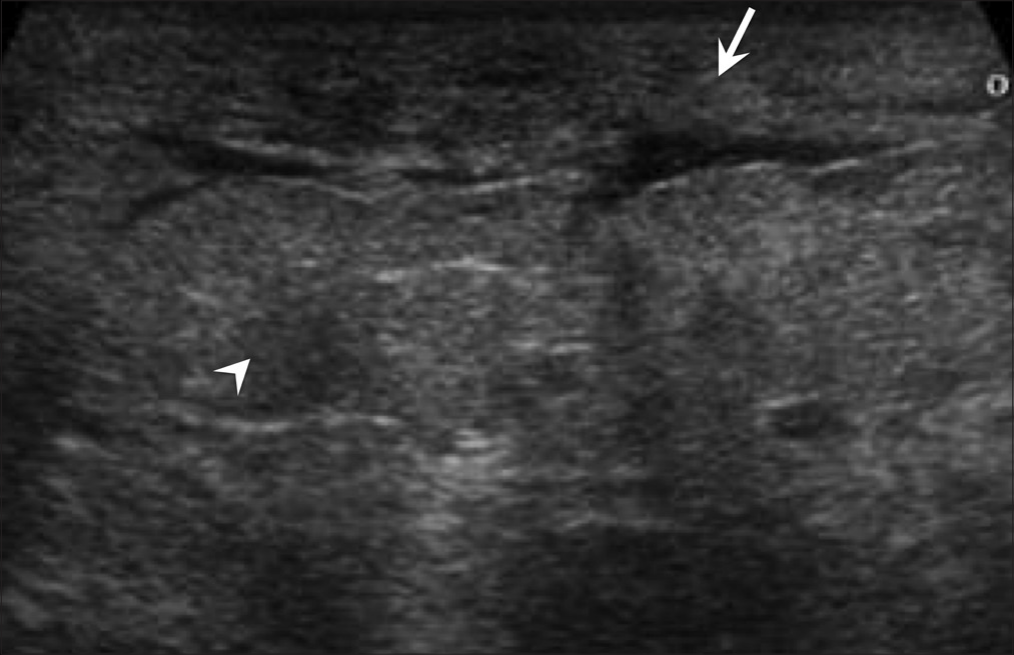
Edema. Breast USG shows skin thickening (arrow) and subcutaneous edema (arrowhead) with a generalized “ground glass” appearance of the breast parenchyma, secondary to enlarged infected left axillary lymph nodes

Edema of the breast can occur following surgery or radiation. It may also occur due to lymphatic or venous obstruction [Figure 9].

## Radial Scar [Figure 10]

**Figure 10 (A,B):**
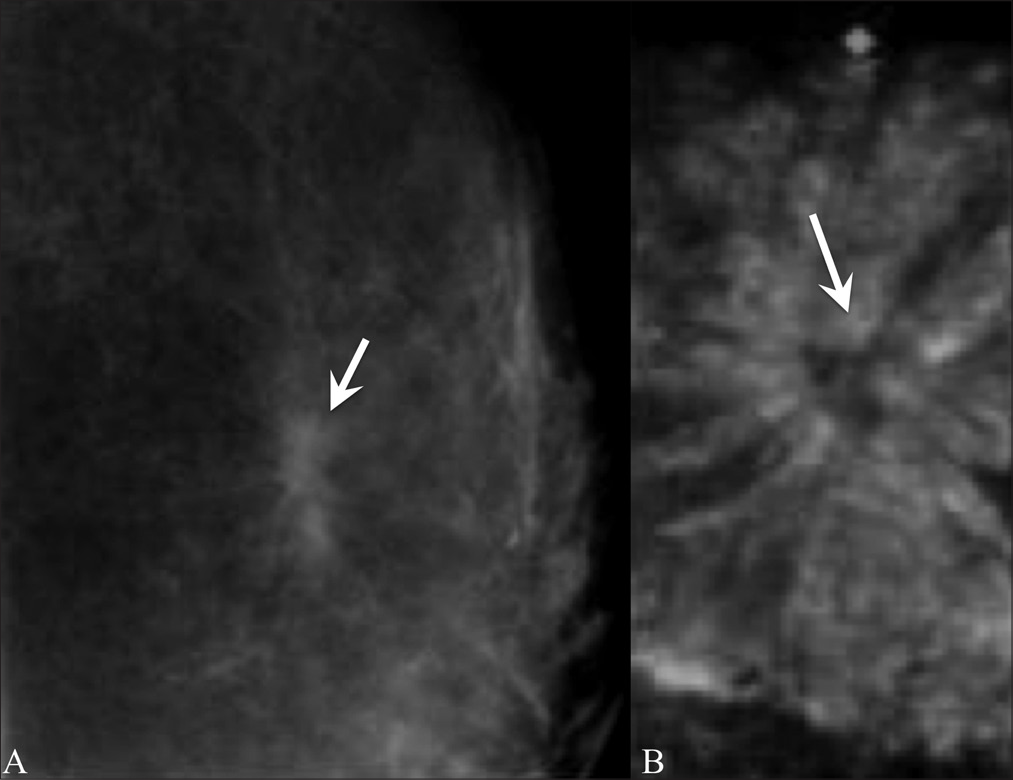
Radial scar. A 44-year-old lady came for routine screening examination. Mammogram (A) showed an ill-defined, irregular spiculated mass (arrow) in the left superior quadrant. A targeted 2D breast USG of the left superior quadrant revealed no significant abnormality other than slightly echogenic soft tissue thickening. However, a 3D multiplanar USG (B) shows a spiculated lesion (arrow) with no central mass, seen only in the coronal plane. A radial scar was diagnosed and confirmed on excision biopsy

This is a benign complex lesion of sclerosing adenosis that appears spiculated on mammography [Figure 10A] and may show a retraction pattern on 3D coronal USG [Figure 10B]. It is usually ill-defined and hypoechoic on USG and may show posterior shadowing.

## Lipomas and Oil Cysts [Figure 11]

**Figure 11 (A,B):**
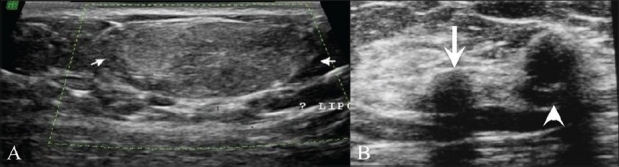
Lipoma (A) and oil cyst (B). Breast USG (A) shows a well-defined, oval, echogenic, mass (arrows) seen superficially in the breast, compressible on probe pressure, suggestive of a lipoma. The appearance of a lipoma varies on breast USG, depending on its location in the breast. It can appear hypoechoic, isoechoic, or echogenic. Breast USG (B) shows an oil cyst (arrow), completely anechoic on USG with peripheral calcifications (arrowheads) showing posterior shadowing

These are fatty tumors in the breast and vary in echogenicity, ranging from echogenic [Figure 11A] lipomas to completely anechoic [Figure 11B] oil cysts.

## Hamartomas or Fibroadenolipomas [Figure 12]

**Figure 12:**
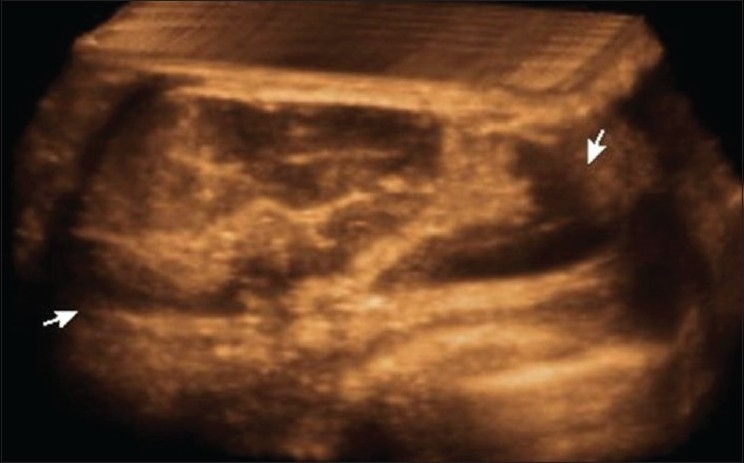
Hamartoma/fibroadenolipoma. 3D USG in a young patient shows a soft, well-circumscribed tumor (arrows) with mixed echogenic and hypoechoic areas

These are fat-containing, soft, benign tumors in the breast, with varying amount of fibrous tissue. On USG, they are heterogeneous with hypoechoic and echogenic areas within them.

## Ductal Carcinoma *In Situ* [Figure 13]

**Figure 13 (A,B):**
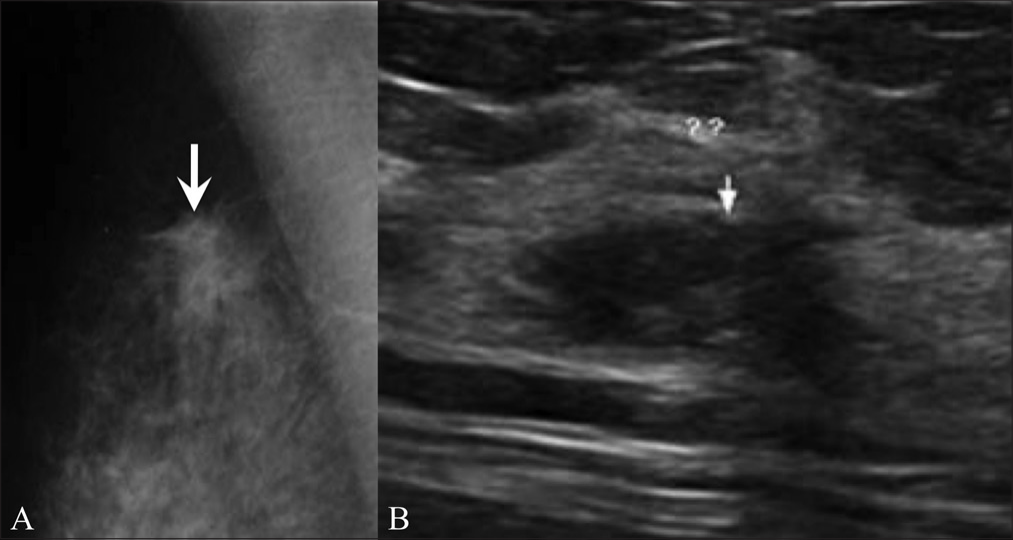
Ductal carcinoma *in situ*. Routine screening mammogram (A) shows a small asymmetric density, which persisted (arrow) in the spot compression view. Targeted breast USG (B) shows a small, ill-defined, hypoechoic mass (arrow) with partially irregular and angular margins. USG-guided core biopsy revealed ductal carcinoma *in situ*

Ductal carcinoma *in situ* may or may not be seen on USG. It may appear as a small mass or as an ill-defined hypoechoic lesion, with echogenic foci within due to microcalcifications [Figure 13].

## Invasive Ductal Carcinoma [Figure 14]

**Figure 14:**
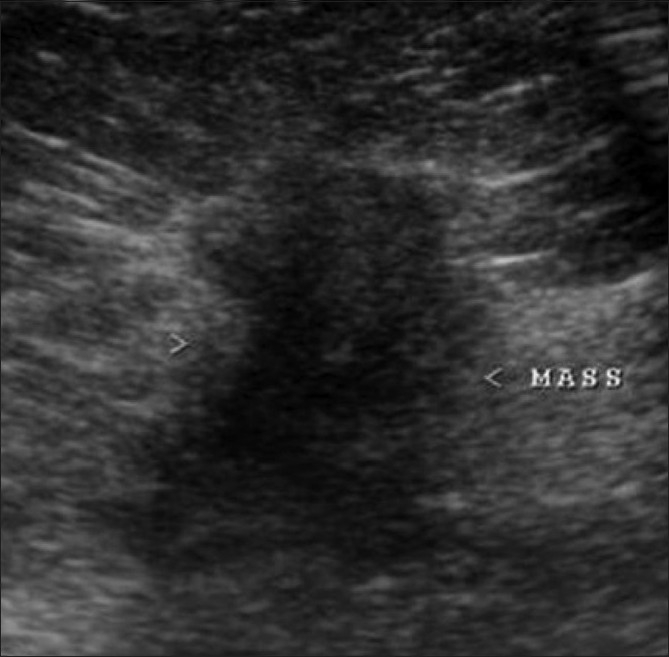
Invasive ductal carcinoma. Breast USG shows an ill-defined, microlobulated, “taller-than-wide” mass (arrows), with marked posterior acoustic shadowing and a thick echogenic rim

These are usually irregular, ill-defined, or microlobulated, and show posterior shadowing. They may be taller than wide [Figure 14] and show a retraction pattern on 3D coronal imaging [Figure 3]. Microcalcifications may be seen as echogenic foci within the lesion.

## Invasive Lobular Carcinoma [Figure 15]

**Figure 15:**
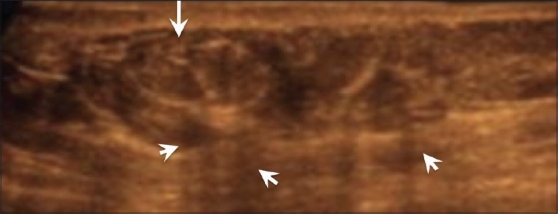
Invasive lobular carcinoma. In a 75-year-old lady with a long-standing history of a palpable lump in the breast, mammograms revealed an ill-defined area of increased density in the left lateral quadrant. Breast USG shows a large, ill-defined, isoechoic mass (arrow) with picket-fence shadowing (arrowheads). USG-guided core biopsy confirmed the diagnosis

This is the second most common breast malignancy and may be seen in elderly women. It is often missed on mammography. On USG, its appearances are variable, ranging from lesions similar to ductal carcinomas to barely visualized areas of architectural distortion with picket-fence shadowing [Figure 15]. Some of these tumors may be occult on USG.[9]

## Medullary Carcinoma [Figure 16]

**Figure 16:**
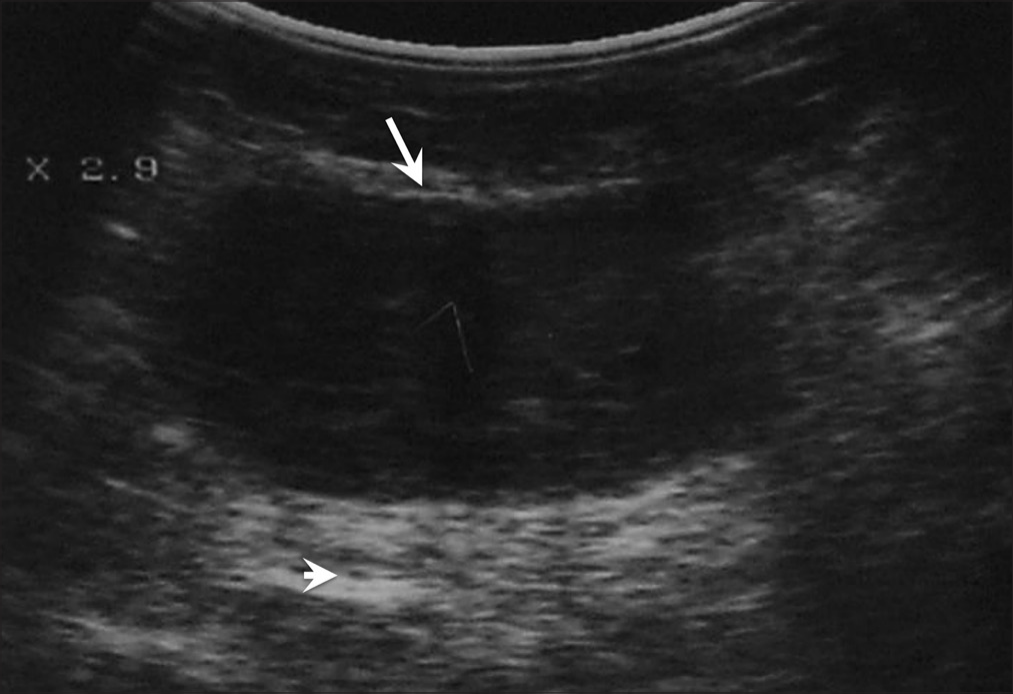
Medullary carcinoma. Breast USG shows a hypoechoic, well-circumscribed mass (arrow) with posterior enhancement (arrowhead)

These are uncommon, benign-appearing lesions, which may be homogenous, hypoechoic, and well-circumscribed on USG [Figure 16].

## Mucinous Carcinoma [Figure 17]

**Figure 17 (A,B):**
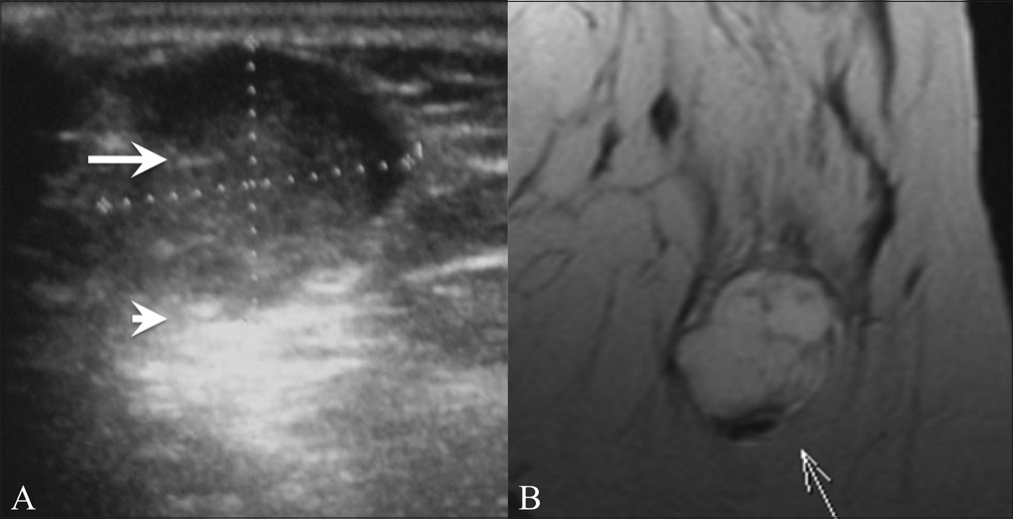
Mucinous carcinoma. It is well-circumscribed on USG(A) with echogenic internal contents (arrow) and posterior enhancement (arrowhead). (B) The mucin shows increased intensity (arrow) on a T2W MRI image

These are also uncommon and benign-appearing. The mucin within may be echogenic on USG and the lesion may show posterior enhancement.

## Paget's Disease [Figure 18]

**Figure 18 (A,B):**
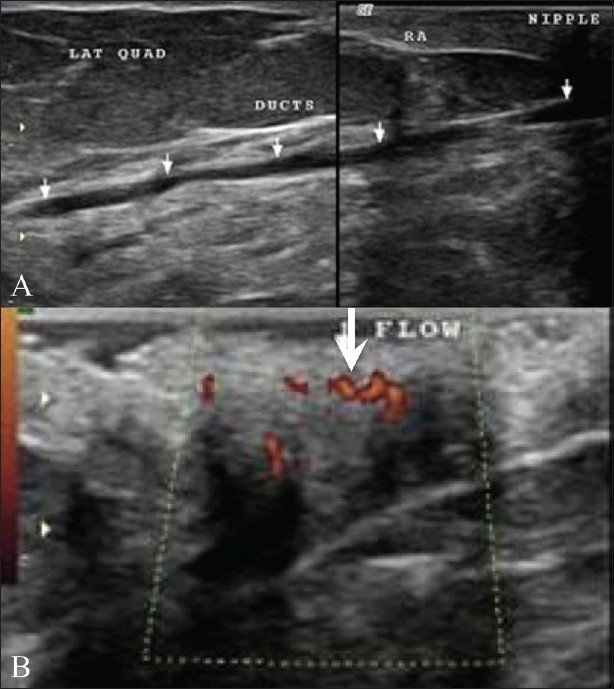
Paget's disease. A 53-year-old lady presented with excoriation of the right nipple and no palpable lump. Mammograms showed no significant findings. Breast USG (A) shows a prominent duct (arrows) in the lateral quadrant. Increased vascularity (arrows) is seen on color Doppler examination (B) in the subareolar region. A clinical diagnosis of Paget's disease was made, which was confirmed on skin biopsy. The patient subsequently underwent a mastectomy

This is a form of ductal carcinoma involving the epidermis, affecting mainly the nipple, areola, and the surrounding region. Mammography and USG may even be normal. MRI may be useful to determine the extent of the disease. Diagnosis is done by skin biopsy [Figure 18].

## Inflammatory Carcinoma [Figure 19]

**Figure 19 (A-D):**
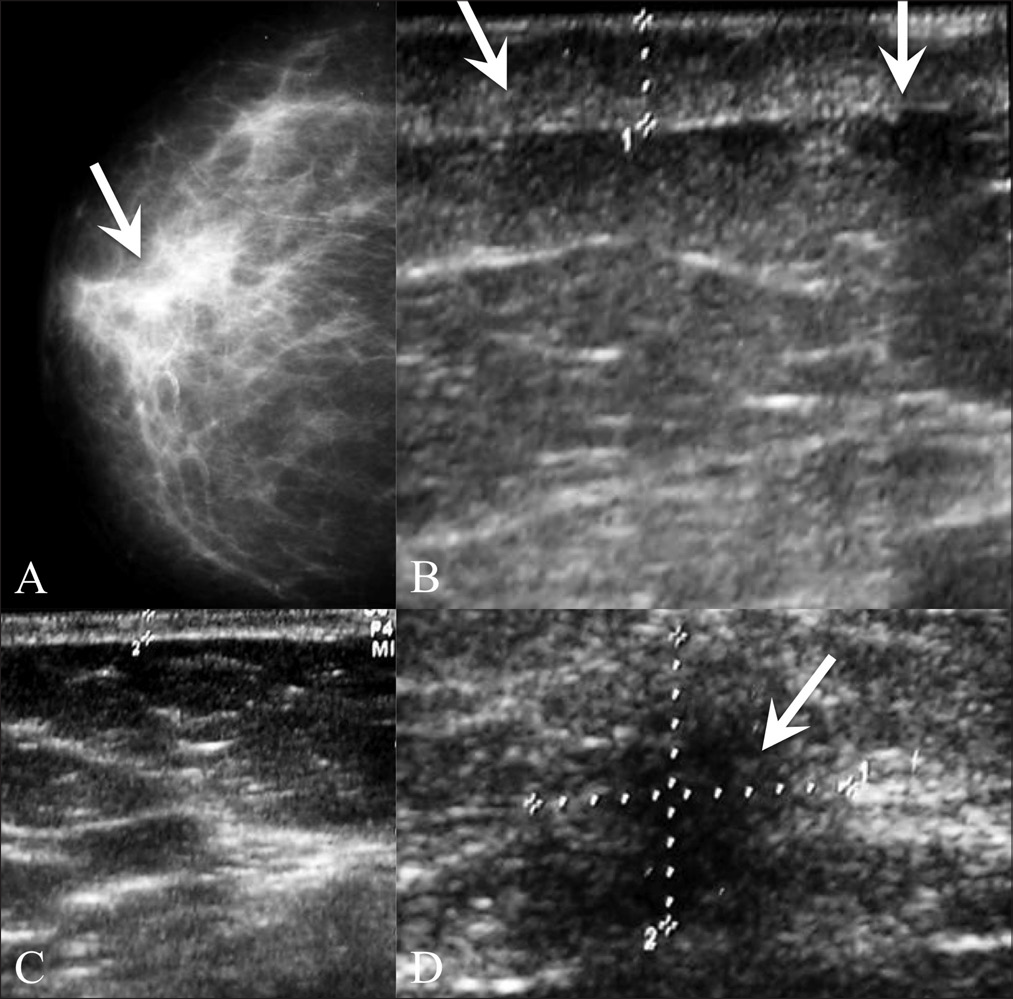
Inflammatory carcinoma. Mammogram (A) reveals increased density (arrows) of the right breast. USG of the right breast (B,D) not only reveals the skin thickening and edema (arrows in B) of the right breast as compared to the normal left breast [C], but also shows the malignant mass [arrow in D]. This was a case of inflammatory breast cancer

This is an aggressive form of breast cancer where the cancer is more diffuse, clogging the lymphatic system under the skin. It is often mistaken for mastitis as the symptoms are very similar and because sometimes there is partial resolution after a course of antibiotics. Mammograms show increased density of the affected breast. MRI may be better for diagnosis. USG shows skin thickening, edema [Figure 19], and enlarged lymph nodes. Core biopsy of the lymph nodes or of the skin may help in diagnosis.

## Recurrent Breast Cancer [Figure 20]

**Figure 20:**
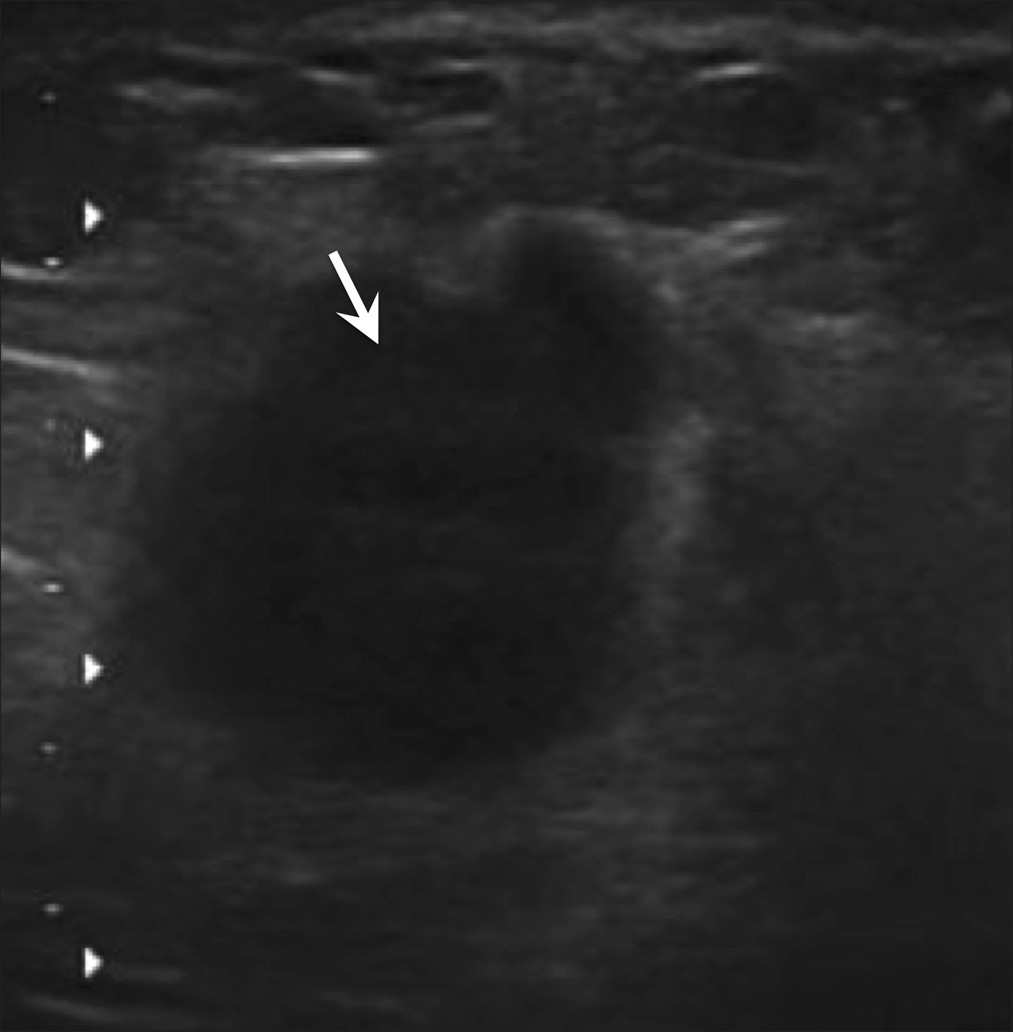
Recurrent breast carcinoma. In this patient with a history of right mastectomy performed 5 years back for a malignant mass and a palpable lump on the right chest wall, USG of the chest wall reveals a well-defined, microlobulated, taller-than-wide mass (arrow), suggestive of recurrence

Recurrence may occur even years after treatment of the primary breast cancer. It may occur in the residual breast or even in the chest wall following mastectomy [Figure 20].

## Breast Implants [Figure 21]

**Figure 21 (A-C):**
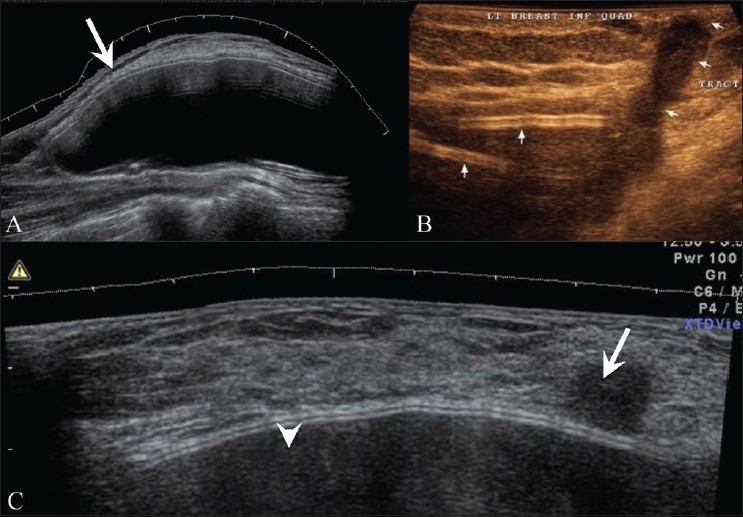
USG (A) of a normal breast implant on extended field of view shows smooth margins and a well-defined capsule (arrows). In a 25-year-old patient with bilateral silicone breast implants with a discharging sinus in the left inferior quadrant, breast USG (B) shows a “step ladder” pattern (arrow) suggestive of intracapsular rupture, along with extracapsular rupture and extravasation of the silicone. Breast USG (C) performed in a lady with breast implants, who refused a mammogram, with a doubtful, palpable lump, an irregular hypoechoic lesion (arrow) is seen abutting the implant (arrowhead). Excision showed malignancy

MRI is more accurate in evaluation of breast implants and implant-related complications. The intact implant has smooth margins and may show some undulations as well as minimal peri-implant fluid [Figure 21A]. An echogenic capsule is seen, forming a triple line surrounding the completely anechoic implant. Rupture may give rise to multiple, linear echogenic lines in the implant – forming a step-ladder pattern [Figure 21B] – and silicone lying outside the implant may give rise to the snow-storm sign of extracapsular rupture. There is no increased incidence of breast cancer in patients with implants [Figure 21C], but it may be difficult to detect in the presence of an implant.

## Gynecomastia [Figure 22]

**Figure 22 (A,B):**
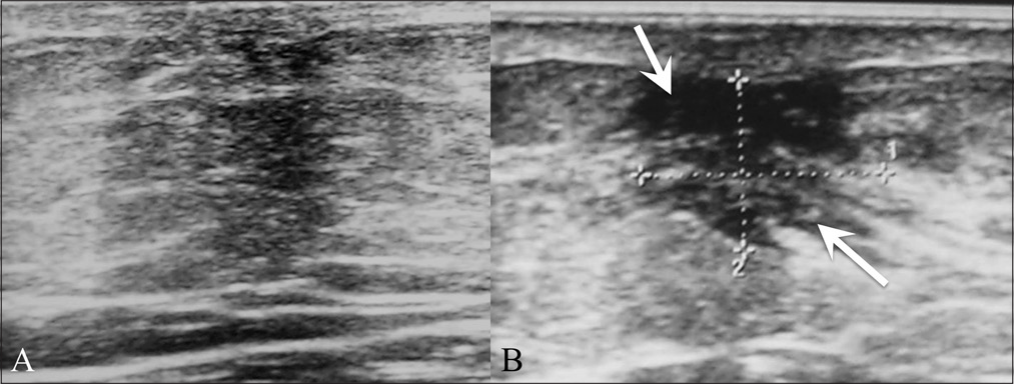
Gynecomastia. In this male patient, breast USG (A) of the right retroareolar region shows only fatty tissue, while the left retroareolar region (B) shows an ill-defined, hypoechoic area of glandular parenchyma (arrows), typical of gynecomastia

In the male breast, gynecomastia is more common than malignancy. It is seen as an ill-defined hypoechoic swelling behind the nipple, appearing similar to glandular tissue in the female breast [Figure 22].

## Male Breast Cancer [Figure 23]

**Figure 23 (A,B):**
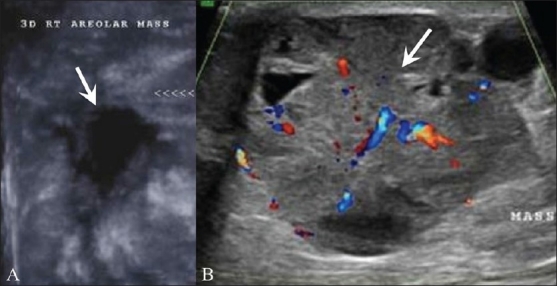
Male breast cancer. In a 80-year-old male patient with a palpable lump in the right breast, a 3D breast USG in the coronal plan (A) reveals a hypoechoic mass (arrow) with multiple spiculations, which was confirmed as an invasive ductal carcinoma on USG-guided core biopsy. In this 68-year-old male patient with a large palpable lump in the left breast, USG (B) with color Doppler shows a well-defined, vascular, heterogeneous mass (arrow) with a few cystic areas within it. USG-guided core biopsy of the mass confirmed the diagnosis of a papillary carcinoma

About 1% of all breast cancers occur in males. USG findings are similar to those of female breast cancer [Figure 23].

## Multifocal Breast Cancer [Figure 24]

**Figure 24:**
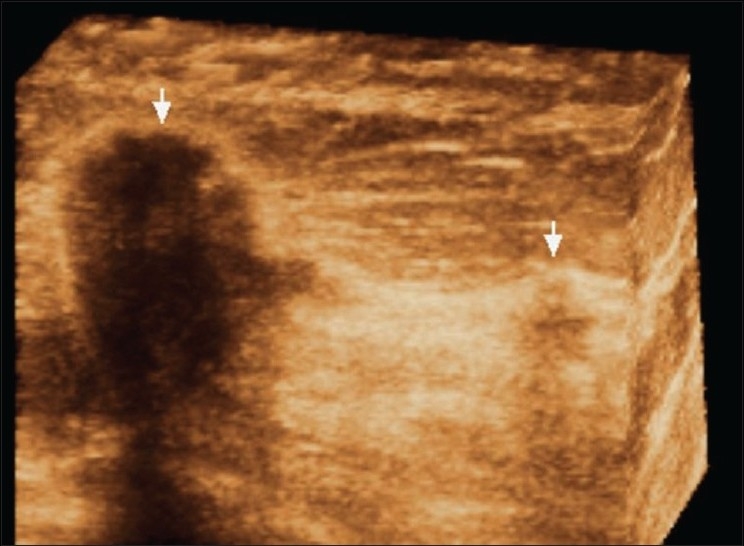
Multifocal cancer. Three-dimensional breast USG shows two foci of breast cancer (arrows)

Breast cancer can quite often be multifocal [Figure 24], multicentric, or even bilateral. Lobular carcinomas are more notorious for being mulifocal.

USG considerably improves the visualization of tumors in radiodense breasts. It improves the specificity of mammography, and when used to complement mammography, it adds more value to the diagnosis. With a cross-sectional imaging technique, tissue visualization free from overprojection is possible. Contour analysis, exact size, and internal tissue composition of tumors can be evaluated. Lesions located in the breast periphery or close to the chest wall can be studied better.

## References

[1] Stavros AT, Thickman D, Rapp CL, Dennis MA, Parker SH, Sisney GA (1995). Solid breast nodules: Use of sonography to distinguish between benign and malignant lesions. Radiology.

[2] Berg WA, Blume JD, Cormack JB, Mendelson EB, Lehrer D, Böhm-Vélez M (2008). Combined screening with USG and mammography vs.mammography alone in women at elevated risk of breast cancer. JAMA.

[3] Weismann C, Hergan K (2007). Current status of 3D/4D volume ultrasound of the breast. Ultraschall Med.

[4] Mendelson EB, Berg WA, Merritt CR (2001). Toward a standardized breast USG lexicon, BI-RADS: USG. Semin Roentgenol.

[5] Weinstein SP, Conant EF, Orel SG, Zuckerman JA, Bellah R (2000). Spectrum of US findings in pediatric and adolescent patients with palpable breast masses. Radiographics.

[6] Berg WA, Campassi CI, Ioffe OB (2003). Cystic lesions of the breast: Sonographic-pathologic correlation. Radiology.

[7] Sklair-Levy M, Sella T, Alweiss T, Craciun I, Libson E, Mally B (2008). Incidence and management of complex fibroadenomas. AJR Am J Roentgenol.

[8] Bassett LW (2000). Imaging of breast masses. Radiol Clin North Am.

[9] Butler RS, Venta LA, Wiley EL, Ellis RL, Dempsey PJ, Rubin E (1999). Sonographic evaluation of infiltrating lobular carcinoma. AJR Am J Roentgenol.

